# Atrial Fibrillation: A Review of Recent Studies with a Focus on Those from the Duke Clinical Research Institute

**DOI:** 10.1155/2014/901586

**Published:** 2014-08-19

**Authors:** Meena P. Rao, Sean D. Pokorney, Christopher B. Granger

**Affiliations:** Cardiology Division, Duke University Hospital, 2301 Erwin Road, DUMC 3845, Durham, NC 27710, USA

## Abstract

Atrial fibrillation is the most common arrhythmia and accounts for one-third of hospitalizations for rhythm disorders in the United States. The prevalence of atrial fibrillation averages 1% and increases with age. With the aging of the population, the number of patients with atrial fibrillation is expected to increase 150% by 2050, with more than 50% of atrial fibrillation patients being over the age of 80. This increasing burden of atrial fibrillation will lead to a higher incidence of stroke, as patients with atrial fibrillation have a five- to sevenfold greater risk of stroke than the general population. Strokes secondary to atrial fibrillation have a worse prognosis than in patients without atrial fibrillation. Vitamin K antagonists (e.g., warfarin), direct thrombin inhibitors (dabigatran), and factor Xa inhibitors (rivaroxaban and apixaban) are all oral anticoagulants that have been FDA approved for the prevention of stroke in atrial fibrillation. This review will summarize the experience of anticoagulants in patients with atrial fibrillation with a focus on the experience at the Duke Clinic Research Institute.

## 1. Introduction

Atrial fibrillation (AF) is the most common arrhythmia and accounts for one-third of hospitalizations for rhythm disorders in the United States [1]. The prevalence of AF averages 1% and increases with age, such that 10% of the population over the age of 80 has AF, and approximately 70% of cases of AF are in patients between 65 and 85 years of age [2]. With the aging of the population, the number of patients with AF is expected to increase 150% by 2050, with more than 50% of AF patients being over the age of 80 [3–8]. This increasing burden of AF will lead to a higher incidence of stroke, as patients with AF have a five- to sevenfold greater risk of stroke than the general population [9–11]. Strokes secondary to AF have a worse prognosis than in patients without AF [12, 13]. Moreover, AF is an independent risk factor for mortality as seen in the Framingham population with an adjusted odds ratio of 1.5 in men and 1.9 in women [14]. Vitamin K antagonists (e.g., warfarin), direct thrombin inhibitors (dabigatran), and factor Xa inhibitors (rivaroxaban and apixaban) are all oral anticoagulants that have been FDA approved for the prevention of stroke in AF. Edoxaban is another factor Xa inhibitor that is likely to be FDA approved in the coming months. In this paper, we will not cover the edoxaban experience in the Effective Anticoagulation with Factor Xa Next Generation in Atrial Fibrillation-Thrombolysis in Myocardial Infarction 48 (ENGAGE AF-TIMI 48) trial in detail given that the primary results have just recently been published.

There has been an explosion of data emanating from these trial databases and from registries over the past 5 years which is redefining much of the knowledge around antithrombotic therapy for AF. This paper will summarize the clinical research experience with anticoagulation in patients with AF at Duke Clinical Research Institute and related work by others.

## 2. Risk Stratification Using Biomarkers

The CHADS2 and CHA2DS2-VASc scores are the primary tools currently used to calculate risk of stroke in patients with AF for the purpose of deciding who has sufficient risk to warrant oral anticoagulation. Although these risk stratification tools are easy to use, they only have a modest discriminating value for individual patients. For example, the CHA2DS2-VASc score has a C-statistic of 0.55–0.64, where 1.0 would represent the perfect ability to correctly rank risk and 0.5 would indicate correct classification only half the time or random chance [15]. Given only a modest ability to discriminate the risk of stroke given available predictive factors, it is important to continue to search for new predictors of risk. There is increasing evidence to support risk stratification with the use of biomarkers for myocardial injury, cardiovascular hemodynamic stress, renal dysfunction, coagulation, and inflammation. Blood biomarkers for these conditions have an independent association with clinical events in AF and may improve risk stratification [16]. Much of this work has been led by investigators at Uppsala Clinical Research Institute and some of it in collaboration with investigators at Duke.

While the mechanism of cardiac troponin elevation in patients with AF is not entirely understood, detecting an elevated level of cardiac troponin has been associated with an increased risk of stroke or systemic embolism and of cardiovascular death. This was first reported in a large data set in the Randomized Evaluation of Long-term Anticoagulant Therapy (RE-LY) biomarker study and subsequently confirmed in the Apixaban for Reduction in Stroke and Other Thromboembolic Events in Atrial Fibrillation (ARISTOTLE) biomarker study [17, 18], with these two trials including a total of 21,081 patients in the biomarker substudies. While RE-LY and ARISTOTLE used troponins I and T, respectively, both studies confirmed that having an elevated troponin level (troponin I > 0.04 ug/L and high sensitive troponin T > 13 ng/L, based on the 99th percentile upper reference limit for healthy subjects) was associated with increased rates of thromboembolic events and cardiovascular death. This relationship was independent of clinical characteristics and other biomarkers, which suggests that troponin measurements may improve the accuracy of risk stratification in AF.

In states of cardiovascular hemodynamic stress, the neurohormone B-type natriuretic peptide (BNP) is secreted from myocytes and BNP or its inactive N-terminal fragment (NT-proBNP) can be detected in the serum. In AF it is hypothesized that elevated levels of natriuretic peptide may be from diastolic dysfunction, leading to increased atrial stress, risk for thrombus formation, and chance of subsequent thromboembolic events. This hypothesis is a plausible mechanism for the association between rising levels of NT-proBNP and increased risk of thromboembolic events and cardiovascular mortality in the RE-LY biomarker study [17]. In this study, the addition of NT-proBNP to clinical risk assessment tools improved the discrimination performance. The results of the RE-LY study were confirmed in the ARISTOTLE biomarker study, showing improvement in risk stratification for overall stroke, as well as for ischemic and for hemorrhagic subtypes, with the addition of NT-proBNP to clinical risk assessment tools [18]. These studies identify the opportunity to use natriuretic peptides as prognostic markers in AF.

Reduced renal function can be measured by calculations of glomerular filtration rates (GFR), based on creatinine, or by cystatin C, which is a small protein that is freely filtered by the glomerulus. Patients in ARISTOTLE showed an inverse association between GFR and rates of stroke and bleeding [19]. In the same population, rising cystatin C levels were independently associated with increased rates of stroke or systemic embolism, mortality, and major bleeding. When added to current risk tools, cystatin C improved risk stratification. Similar findings with cystatin C were identified in the RE-LY biomarker study [20]. While cystatin C had improved risk stratification for stroke, GFR was a better predictor of bleeding, so both markers have the potential to improve risk prediction in patients with AF.

D-dimer, a marker of coagulation in AF, may also be a clinically useful predictor of risk. A significant association between baseline D-dimer levels and risk of stroke, cardiovascular death, and major bleeding was seen in the RE-LY biomarker study and confirmed in the larger ARISTOTLE data set [17, 18].

Inflammation may be involved in the development and the perpetuation of AF, and markers of inflammation may have prognostic significance [21]. Markers of inflammation that have been studied in AF include interleukin-6 (IL-6) and C-reactive protein (CRP). Initially, a small study reported an association between IL-6 and a composite outcome of stroke and death [22]. A larger study subsequently found an association between CRP and all-cause mortality, in addition to a composite endpoint of TIA/stroke, systemic embolism, acute coronary syndrome, acute heart failure, and cardiac death [23]. Finally, the RE-LY biomarker study showed an independent association between IL-6 and stroke or systemic embolism, while CRP levels were associated with cardiovascular mortality after multivariate adjustments [17]. These studies indicate that there may be potential to use markers of inflammation in risk stratification for AF, but further investigation will need to be done prior to inclusion as a routine marker.

## 3. Anticoagulation with Warfarin in High-Risk Patients

**Figure 1:**
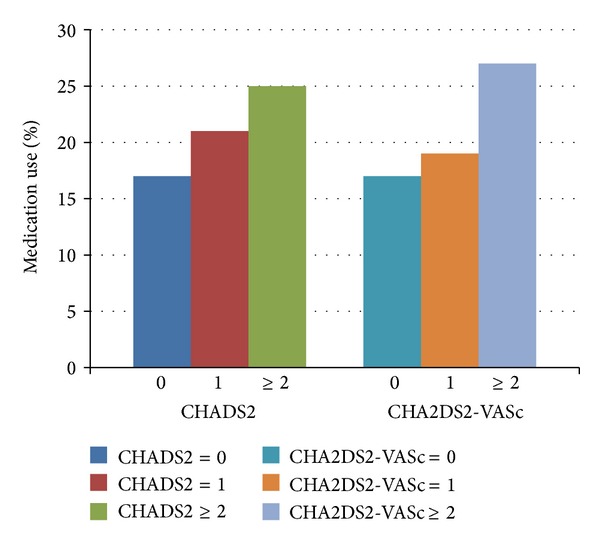
Duke Databank. Warfarin use at discharge following acute myocardial infarction according to CHADS2 and CHA2DS2-VASc scores [24]. Percentages of medication use were visually estimated from primary publication.

Patients with AF and acute myocardial infarction (AMI) are at high risk of death and major cardiovascular outcomes. In the Duke Databank for Cardiovascular Disease, patients with AMI and AF, who underwent cardiac catheterization during their AMI hospitalization between 1995 and 2007, tended to be older, have increased comorbidities, and have higher one-year mortality (24). Given the increase in comorbidities, this population had a higher stroke risk based on CHADS2 and CHA2DS2-VASc scores, as well as increased bleeding risk based on the ATRIA bleeding score. The identified population had a low rate of warfarin use with less than 25% of patients discharged on warfarin, and the rate of warfarin use was not correlated with stroke or with bleeding risk (Figure 1). The rate of warfarin use remained low over time, despite reductions in clopidogrel use from 6 to 12 months after discharge. These findings suggest that providers are using factors other than clinical risk stratification tools to guide anticoagulation decisions in high-risk patients. Providers may also be hesitant to prescribe triple therapy (therapy with aspirin, clopidogrel, and warfarin) because of bleeding risk [24]. It is important to note that the current guidelines on anticoagulation in the setting of stent placement were not available at the time these patients were followed.

**Figure 2:**
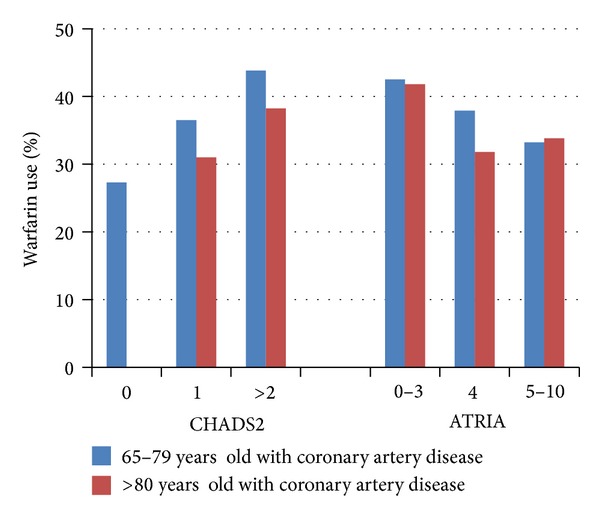
Duke Databank. Warfarin use at discharge among patients with coronary artery disease according to CHADS2 (stroke) and ATRIA (bleeding) scores in patients 65 to 79 versus ≥80 years of age [25].

Another high-risk group of patients that we have studied is older patients with AF and coronary artery disease. Choosing the most appropriate antithrombotic regimen in this group is challenging, given the increased risk for bleeding related to both age and concomitant antiplatelet therapy. In order to guide clinicians, the experience of different antithrombotic regimens in older patients with cardiovascular disease was described, using patients from the Duke Databank for Cardiovascular Disease [25]. The study included patients with AF, aging greater than 65 years, and angiographically confirmed coronary artery disease between 2000 and 2010. Analysis of this group of patients showed that the use of warfarin was concordant with patient risk, such that there was increased use with higher stroke risk (defined by CHADS2 and CHA2DS2-VASc scores) and decreased use with higher bleeding risk (using the ATRIA score). However, among patients ≥80 years old, the rate of warfarin use was <40%, while 92% of these patients were at moderate to high risk of stroke [26]. This shows underuse of anticoagulation in patients ≥80 years old (Figure 2).

It is important to understand if underuse of warfarin therapy in the elderly is due to appropriate reasons that may include the risk of bleeding and falls. Providers may be hesitant to start anticoagulation with warfarin in the elderly because of concerns over bleeding risk and greater comfort in using aspirin alone [27–29]. There is an association between lower stroke and lower bleeding risk with better INR control as reported from the Stroke Prevention Using ORal Thrombin Inhibitor in Atrial Fibrillation (SPORTIF) III and V studies [30]. Optimal warfarin outcomes have been consistently found to occur with good INR control [26]. Warfarin is far more effective than either aspirin or the combination of aspirin and clopidogrel and has comparable bleeding rates [31–33]. Thus, antiplatelet therapy should only be used in patients who refuse anticoagulation and perhaps in patients at very low risk of stroke. The AVERROES trial showed that apixaban was far more effective (46% relative risk reduction in stroke) and nearly as safe (nonsignificant 12% increase risk of major bleeding and no difference in ICH) as aspirin [34]. Fall risk in elderly patients on antithrombotic therapy was studied in a meta-analysis, which demonstrated that elderly patients taking warfarin would have to fall approximately 300 times per year for the risk of bleeding complications from falling to outweigh the benefits of embolic stroke prevention [35]. Moderate risk of falling in the elderly population should not be an absolute contraindication to anticoagulation [27, 36].

Warfarin continues to be underused, particularly in the high-risk populations, despite the evidence that the benefit from reduction of thromboembolic events outweighs bleeding risk.

## 4. Novel Oral Anticoagulants

There have been four randomized clinical trials comparing novel oral anticoagulants to warfarin in nonvalvular AF: RE-LY, Rivaroxaban Once Daily Oral Direct Factor Xa Inhibition Compared with Vitamin K Antagonism for Prevention of Stroke and Embolism Trial in Atrial Fibrillation (ROCKET-AF), ARISTOTLE, and ENGAGE AF-TIMI 48. These oral anticoagulants have been approved by the Federal Drug Administration for stroke prevention in patients with AF. In this review, we will focus on a summary of trial results, drug pharmacokinetics and pharmacodynamics, and considerations in high-risk patient populations (Tables 1, 2, and 3) [37]. As mentioned previously, ENGAGE AF-TIMI 48 trial, completed by the TIMI Investigators, will not be covered in detail.

### 4.1. Trial Summaries and Drug Descriptions

The RE-LY trial was an open label randomized trial, which studied dabigatran, a direct thrombin inhibitor. Dabigatran has 80% renal excretion and its bioavailability decreases by 20% with proton pump inhibitor use [38]. The RE-LY trial demonstrated that dabigatran 150 mg twice daily was superior to warfarin for the prevention of stroke (ischemic and hemorrhagic) and systemic embolism [39]. There were similar rates of major bleeding between the two agents, but dabigatran had higher rates of gastrointestinal bleeding. The 110 mg twice-daily dose was found to be noninferior to warfarin for stroke or systemic embolism but had lower rates of major bleeding. Both doses reduced rates of intracranial hemorrhage by over two-thirds.

ROCKET-AF was a randomized, double blind, and double dummy trial, which found that rivaroxaban was noninferior to warfarin for the primary endpoint of prevention of stoke or systemic embolism in patients with CHADS2 score > 2, as well as for major bleeding [40]. Patients receiving rivaroxaban had a lower risk of hemorrhagic stroke and intracranial bleeding compared with warfarin. At baseline, ROCKET-AF had three times as many patients with CHADS2 scores 3–6 as RE-LY and ARISTOTLE, resulting in a higher overall risk for thromboembolic events. Rivaroxaban is a direct factor Xa inhibitor and has 36% renal excretion [41]. Patients taking medications affecting the CYP3A4 pathway (ketoconazole, clarithromycin, erythromycin, rifampin, and protease inhibitors) were excluded from the ROCKET-AF trial due to potential drug-drug interactions [37].

ARISTOTLE, similar to ROCKET-AF, was a randomized, double blind, and double dummy trial, which compared apixaban to warfarin and found that apixaban was superior to warfarin for the endpoints of stroke (ischemic and hemorrhagic) and major bleeding [42]. Patients receiving apixaban instead of warfarin had lower rates of intracranial bleeding and decreased mortality. Apixaban has 27% renal excretion. Similar to ROCKET-AF, patients taking medications affecting the CYP3A4 pathway were excluded from ARISTOTLE for potential drug-drug interactions [43].

### 4.2. Considerations in High-Risk Patient Populations

#### 4.2.1. Impaired Renal Function

Patients with AF and renal impairment are at high risk for thromboembolism and also for bleeding. Given the higher drug concentrations of the novel anticoagulants with decreased renal excretion, it is important to consider the risk of bleeding in this high-risk population. Since dabigatran has the highest renal clearance of 80%, apixaban and rivaroxaban may be better options in patients with severe renal dysfunction [37]. Patients with a GFR < 30 mL/min were excluded from ROCKE-AF and RE-LY [40, 44]. In ROCKET-AF, rivaroxaban 20 mg daily was studied in patients with a GFR ≥ 50 mL/min and a lower dose of 15 mg was studied in patients with moderate renal impairment (GFR 30–49 mL/min). The lower dose of rivaroxaban in patients with moderate renal impairment showed noninferiority to warfarin therapy in a subgroup analysis [45]. In ARISTOTLE, patients with a GFR of <25 mL/min or a creatinine > 2.5 mg/dL were excluded. Patients with two of the following criteria were given a decreased dose of 2.5 mg twice daily: age ≥ 80, creatinine ≥ 1.5 mg/dL, and weight ≤ 60 kg [42]. A subgroup analysis of ARISTOTLE showed a consistent effect for both efficacy and safety across the spectrum of renal function. Given that patients with severe renal impairment were excluded in the trials, warfarin should still be used for anticoagulation in those patients.

#### 4.2.2. Prior Stroke or TIA

Patients with previous stroke or TIA are at higher risk of major bleeding and stroke or systemic embolism [46]. Looking at the three novel anticoagulants in this high-risk population, there were consistent effects of the novel oral anticoagulants versus warfarin, when comparing rates of stroke or systemic embolism and major hemorrhage in patients with and without history of TIA or stroke [37]. Each of the novel agents was found to decrease hemorrhagic stroke in patients with a history of previous stroke or TIA. The novel agents should be considered in patients with previous stroke or TIA.

#### 4.2.3. High Stroke or Bleeding Risk

Patients who are at high risk of stroke are identified using the CHADS2 and CHA2DS2-VASc risk classification tools [47, 48]. Similarly, the HAS-BLED score is used to quantify bleeding risk [49]. Since these treatment tools have been used in the past to help guide decisions regarding anticoagulation using vitamin K antagonists and antiplatelet agents, a subgroup analysis of ARISTOTLE was performed to determine the treatment effects of apixaban according to stroke and bleeding [50]. In each trial, the benefits of the novel drugs versus warfarin were consistent across risk groups. A subgroup analysis of ARISTOTLE found that apixaban, compared with warfarin, significantly lowered rates of stroke or systemic embolism across all risk categories for stroke, as well as decreasing major bleeding in all risk categories for bleeding [50]. There was a trend toward greater risk reduction for intracranial hemorrhage among patients with the highest risk of bleeding [46].

#### 4.2.4. Concomitant Antiplatelet Therapy

Antiplatelet therapy was given to about 38% of the population at baseline in the RE-LY trial and about 31% of the trial population in the ARISTOTLE trial [51, 52]. While the treatment effects of the direct oral anticoagulants versus warfarin were consistent with and without background aspirin, the risk of bleeding with aspirin was about 50% higher than without aspirin with both warfarin and the new drugs. Additionally, a substantial portion of the aspirin-treated population had no clear reason (such as vascular disease) to be on the aspirin. Thus, there appears to be a major opportunity to improve safety of oral anticoagulation by avoiding unnecessary aspirin.

#### 4.2.5. Patients with Prior Coronary Disease

Patients with prior coronary disease appear to have consistent treatment effects with the new drugs versus warfarin for both efficacy and safety. It is of note that there was a nonstatistically significant increased risk in myocardial infarction with dabigatran, as compared to warfarin in the overall RE-LY population. This may have been because warfarin is effective in preventing myocardial infarction rather than a hazard of dabigatran [39]. In contrast, when apixaban was used alone without aspirin, there were numerically fewer myocardial infarctions than warfarin [53].

#### 4.2.6. Valvular Disease

Patients with mechanical bileaflet aortic or mitral valves were studied in an open label randomized, blinded endpoint phase II trial that evaluated the safety of dabigatran compared with warfarin, called RE-ALIGN [54]. There were two arms of the study: (1) therapy started during the valve implant hospitalization and (2) therapy started at least 3 months after valve implantation. Both arms were terminated early due to excess thromboembolic and excess bleeding events in the dabigatran arm. There have also been case reports of patients with mechanical valves developing thrombus on the valves shortly after being changed from warfarin to dabigatran [37, 55]. In patients with mechanical aortic or mitral valves, warfarin is still the anticoagulant that should be used.

In contrast, we also know that about one-quarter of patients with AF have moderate or severe valve abnormalities other than mechanical prosthetic valves or clinically significant mitral stenosis. These patients had consistent benefits of apixaban in the ARISTOTLE trial [56]. Thus, “nonvalvular” AF is a misnomer. When used to identify the population for which the new drugs are not suitable this term should be limited to moderate or severe mitral stenosis or mechanical prosthetic valves.

#### 4.2.7. Elderly Patients

Elderly patients with AF have very high risk of thromboembolic events and, given a consistent relative risk reduction with anticoagulation, are a population that has some of the largest benefits from anticoagulation. Despite this benefit, they tend to be undertreated due to concerns of bleeding as mentioned previously. The low risk of intracranial hemorrhage with novel agents may make these medications attractive options for this population. Patients who were older than 75 years were well represented in the oral anticoagulant trials. The treatment benefits were consistent in this population with the one exception being that, with 150 mg of dabigatran, there was relatively more bleeding versus warfarin in the elderly [39]. For apixaban, even for patients ≥ age 80, the benefits of the novel drug were clear and consistent [42]. Thus, the novel oral anticoagulants appear to be a good choice in patients older than 75 years.

#### 4.2.8. Elevated Liver Enzymes

Patients with abnormal liver enzymes at baseline were excluded from the novel oral anticoagulant trials as there was concern given that the first oral direct thrombin inhibitor, ximelagatran, caused liver function abnormalities. While there is therefore uncertainty about risk of initiating these drugs to patients with liver disease, the subsequent novel agents have not caused similar liver function abnormalities.

#### 4.2.9. Prior Warfarin Use

Therapy with vitamin K antagonists (VKA) has a higher risk of intracranial hemorrhage in the first three months of treatment [57]. In ACTIVE-W, the warfarin-naïve population had worse outcome with warfarin and therefore relatively better outcome with the comparator antiplatelet therapy arm [33]. Due to these observations, the RE-LY trial design required that 50% of the patients were VKA-naïve. However, the treatment effect of dabigatran did not differ according to prior VKA use. Similar analyses in the ARISTOTLE and ROCKET-AF trials found that the treatment effects of apixaban or rivaroxaban were also similar in warfarin-naïve and warfarin-experienced populations [58]. These analyses show that whether or not a patient is or has been on warfarin, one can expect the same advantages of the novel agents versus warfarin.

#### 4.2.10. Time in Therapeutic Range

An important set of analyses addresses the question of whether the direct oral anticoagulants perform well against warfarin when there is good INR control. This is relevant since a substantial portion of patients currently on warfarin, particularly in anticoagulation clinics or in countries with systems in place to optimize warfarin use, have a “time in therapeutic range” of INR 2-3 over 65 to 70% of the time. The median time in therapeutic range in the 3 older trials has been 58–66%, and it was 68% in the ENGAGE-AF trial. Careful analyses have defined the treatment effect of the new drugs versus warfarin according to time in therapeutic range for warfarin-treated patients at the site level. While there may be modestly less benefit for stroke prevention at sites with excellent INR control, the treatment effect of the novel agents is relatively consistent among ranges of INR control, including for reduction in ICH compared to warfarin [40, 59, 60]. Thus, while patients on well-controlled warfarin are a lower priority regarding treatment with the new agents, they nevertheless would be expected to benefit.

## 5. Future Studies

### 5.1. Additional Clinical Trials

A series of additional clinical trials have been planned or are underway to refine our understanding of use of the new oral anticoagulants for AF. These include trials in electrical cardioversion and postcoronary stenting and around atrial ablation procedures. Other trials are testing the new drugs for related conditions, including for heart failure and for embolic stroke of undetermined origin, a subset of cryptogenic stroke [61]. Trials to define the use of the novel anticoagulants for subclinical AF, detected with cardiac devices or other methods, are being considered.

### 5.2. Implementation Research

Despite the effectiveness of anticoagulation, recent literature reviews and studies have documented that current practice does not follow published guidelines, with undertreatment resulting in substantial occurrence of preventable ischemic stroke [62]. In a US study, only 50% of eligible elderly patients received anticoagulation, and this gap between guidelines and clinical practice extends globally [63]. A global registry called the RE-LY registry, including 47 countries with 15,174 patients with AF, was created to determine if there were differences in the treatment of AF globally [64]. They found that patients who were eligible for anticoagulation (CHADS2 score ≥ 2) were prescribed anticoagulation only 10–65% globally.

The reasons for underuse of anticoagulation are poorly understood. As the risk of stroke increases, the rate of anticoagulation use is not different or decreases. This may be due to concerns of providers, regarding the risk of bleeding and the risk-benefit tradeoff of treatment for higher-risk populations. However, data suggests that cardiologists and primary care physicians have different conceptualizations of stroke and bleeding risks and primary care physicians may be less likely to prescribe oral anticoagulants [25, 65]. The AVERROES trial provided some insight into reasons for patients were deemed to be “unsuitable for warfarin” with 42% unable to maintain therapeutic INR, 43% unlikely to monitor INR, and 37% refusing warfarin [34]. The uncertainty about the true reasons for nonuse limits our ability to address the gaps in care. Understanding why patients are not being treated is necessary to develop targeted strategies to increase the proportion of patients who are treated worldwide.

Few quality improvement interventions have been evaluated to determine impact on patient care and clinical outcomes for patients with AF with the goal of decreasing underuse of anticoagulation to reduce preventable stroke rates. Using methods that have been shown to improve adherence, including education of health care providers, improved communication between physicians and patients, patient education, and measuring and providing feedback regarding adherence, researchers at the Duke Clinical Research Institute have designed an education intervention aimed to increase the number of patients who are eligible for anticoagulation to receive anticoagulant therapy. To determine if this educational intervention is effective, we have designed an international clustered randomized trial: IMPACT-AF. This trial will start enrolling patients in 2014 and will follow them for one year. Implementation of Demonstration Project for Health Systems, Atrial Fibrillation (INFORM-AF), is a separate initiative underway that we designed to improve treatment for stroke prevention in AF in the United States health systems. In order to develop an effective performance measurement system for quality of treatment of AF, we plan to measure appropriate use and nonuse. We will use qualitative interviewing to develop a system to categorize patients according to reasons for withholding anticoagulation therapy with the goal of defining performance indicators that can be used for quality assessment of AF therapy within health care systems and clinical practices. We will then work with several health systems in the US to implement quality improvement strategies to increase the use of anticoagulation. Based on the information gathered through the interviewing process, a practice toolkit that contains a series of tools that address the gaps and barriers to quality care will be developed and shared with all participants. Health systems will be encouraged to promote the use of the toolkit in daily practice.

The aim of the study is to document and increase the rates of appropriate use of oral anticoagulation and patient adherence to and persistence with the treatment plans. These metrics will be tracked over the course of one year. The education and system-based intervention will be executed over the course of the project. We will prospectively follow the impact of the intervention on clinical outcomes such as admission for stroke, bleeding, and death.

The Outcomes Registry for Better Informed Treatment of Atrial Fibrillation (ORBIT-AF registry) is a national registry that was created to describe the population of patients with AF in the United States with a focus on the optimization of outpatient management. The registry has now completed its three-year follow-up period. The ORBIT-AF registry includes approximately 200 sites and 10,000 patients, who are older than 18 years with AF and the ability to follow up every six months. Many important descriptions of AF patient characteristics in the US and insights regarding care for patients with AF have been described. Hess et al. analyzed the use of evidence based therapies for treating other cardiovascular existing risk factors. More than 93% of patients in the registry were eligible for at least one guideline-based therapy for a coexisting cardiovascular disease, but only 46% of patients received all recommended treatments, highlighting an opportunity to improve care [66]. Cullen et al. focused their analysis on oral anticoagulant use with increasing stroke and bleeding risk. They found that oral anticoagulant use increased with higher CHADS2 scores but fell slightly with increasing ATRIA bleeding risk. CHADS2 stroke risk had a small impact on those with high bleeding risk [67]. Most recently, Steinberg et al. looked into the effect of aspirin therapy with concurrent anticoagulation. They found that major bleeding and bleeding hospitalizations were significantly higher in patients on both oral anticoagulation and aspirin than those patients on oral anticoagulation alone [68]. There are many ongoing analyses based on the data from this registry.

The ORBIT-AF II registry has started to enroll. Similar to ORBIT-AF, ORBIT-AF II is a multicentered prospective outpatient registry; however, this registry will focus on postapproval observational data to evaluate outcomes of novel oral anticoagulant therapies in a broader outpatient setting. This registry started enrollment in 2013 with an estimated completion date in 2017.

## 6. Conclusion

There has been an enormous amount of data generated and analyses performed, including those in collaboration with our research groups at the Duke Clinical Research Institute, regarding oral anticoagulation for preventing stroke in AF. This includes over 75,000 patients enrolled in 5 large trials testing the new direct oral anticoagulants [40, 42, 69, 70] and several large registries enrolling many tens of thousands of patients [64, 68, 71]. The most important observations and discoveries so far have been the undertreatment of oral anticoagulation to prevent stroke, the safety and effectiveness of the new drugs versus warfarin, risk of stroke and of bleeding in various important subgroups, and novel risk factors for these events that may help in guiding most effective treatments. Important ongoing research is addressing how to safely and effectively use the new drugs in general practice and how to more effectively implement oral anticoagulation, including with warfarin, to better prevent AF-related stroke in the US and around the world.

## Figures and Tables

**Table 1:** Baseline characteristics for novel oral anticoagulant trials.

	RE-LY	ROCKET-AF	ARISTOTLE
Study drug	Dabigatran 150 mg twice daily	Rivaroxaban 20 mg daily	Apixaban 5 mg twice daily
Renal dose adjustment	Dabigatran 110 mg twice daily	Rivaroxaban 15 mg daily	Apixaban 2.5 mg twice daily
Average CHADS2 score	2.2	3.5	2.1
Mean age	71	73	70
Creatinine clearance >30, <50 ml/min	19.4%	20.8%	15.2%
Creatinine clearance >50 ml/min	80.6%	79.2%	83.4%
Prior warfarin use	49.6%	62.4%	57.2%
Prior myocardial infarction	16.6%	17.3%	14.2%
Prior stroke/TIA	20.0%	54.8%	19.4%

**Table 2:** Hazard ratios with 95% CI for novel oral anticoagulants compared with warfarin [37].

	RE-LY∗	ROCKET-AF	ARISTOTLE
Stroke and systemic embolism	0.65 (0.52–0.81)	0.88 (0.75–1.03)	0.79 (0.66–0.95)
Ischemic or unspecified stroke	0.76 (0.60–0.98)	0.91 (0.73–1.13)	0.92 (0.74–1.13)
Hemorrhagic stroke	0.26 (0.14–0.49)	0.59 (0.37–0.93)	0.51 (0.35–0.75)
Myocardial infarction	1.27 (0.94–1.71)	0.81 (0.63–1.06)	0.88 (0.66–1.17)
All-cause mortality	0.88 (0.77–1.00)	0.85 (0.70–1.02)	0.89 (0.80–0.998)
Major bleeding	0.93 (0.81–1.07)	1.04 (0.90–1.20)	0.69 (0.60–0.80)
Gastrointestinal bleeding	1.50 (1.19–1.89)	1.47 (1.20–1.81)	0.89 (0.70–1.15)
Intracranial bleeding	0.40 (0.27–0.60)	0.67 (0.47–0.93)	0.42 (0.30–0.58)

∗HR presented are for the 150 mg twice daily dose of dabigatran.

**Table 3:** Hazard ratios with 95% CI for subgroup analyses for novel agents versus warfarin.

	Stroke and systemic embolism	Major and clinically relevant nonmajor bleeding
Renal function	HR (95%CI)	HR (95%CI)

ARISTOTLE		
GFR ≤50	0.79 (0.55–1.14)	0.50 (0.38–0.66)
GFR 50–80	0.74 (0.56–0.97)	0.77 (0.62–0.94)
GFR >80	0.88 (0.64–1.22)	0.80 (0.61–1.04)
RE-LY∗		
Dabigatran 150 mg BID		
CrCl <50 ml/min	0.50 (0.25–0.80)	n/a
CrCl 50–79 ml/min	0.70 (0.48–0.90)	n/a
CrCl ≥80 ml/min	0.78 (0.40–1.25)	n/a
Dabigatran 110 mg BID		
CrCl <50 ml/min	0.77 (0.50–1.20)	n/a
CrCl 50–79 ml/min	0.90 (0.75–1.26)	n/a
CrCl ≥80 ml/min	0.90 (0.60–1.55)	n/a
ROCKET-AF		
CrCl 30–49 ml/min	0.84 (0.57–1.23)	0.98 (0.84–1.14)
CrCl >50 ml/min	0.78 (0.63–0.98)	1.04 (0.96–1.13)

Prior stroke/TIA		

ARISTOTLE		
Prior stroke/TIA	0.76 (0.56–1.03)	1.07 (0.09–2.04)
No prior stroke/TIA	0.22 (0.03–0.47)	0.93 (0.54–1.32)
RE-LY		
Dabigatran 110 mg	RR 0.84 (0.58–1.2)	RR 0.66 (0.48–0.90)
Dabigatran 150 mg	RR 0.75 (0.52–1.08)	RR 1.01 (0.77–1.34)
ROCKET-AF		
Prior stroke/TIA	0.94 (0.77–1.16)	0.96 (0.87–1.07)
No prior stroke/TIA	0.77 (0.58–1.01)	1.10 (0.99–1.21)

Prior warfarin use		

ARISTOTLE		
VKA-experienced	0.73 (0.57–0.95)	0.66 (0.55–0.80)
VKA-naïve	0.86 (0.67–1.11)	0.73 (0.59–0.91)
RE-LY		Major bleeding
Dabigatran 150 mg BID		
VKA-experienced	RR 0.66 (0.48–0.89)	RR 0.40 (0.24–0.67)
VKA-naïve	RR 0.63 (0.46–0.87)	RR 0.46 (0.27–0.78)
Dabigatran 110 mg BID		
VKA-experienced	RR 0.87 (0.66–1.15)	RR 0.32 (0.18–0.56)
VKA-naïve	RR 0.93 (0.70–1.24)	RR 0.27 (0.14–0.52)
ROCKET-AF		
VKA-experienced	0.97 (0.78–1.19)	1.09 (0.99–1.19)
VKA-naïve	0.76 (0.59–0.98)	n/a

Elderly		

ARISTOTLE		Major bleeding
Age ≥75 years	0.71 (0.53–0.95)	0.64 (0.52–0.79)
Age 65–<75	0.72 (0.54–0.96)	0.71 (0.56–0.89)
Age <65	1.16 (0.77–1.73)	0.78 (0.55–1.11)
RE-LY		
Dabigatran 150 mg BID		
Age ≥75 years	0.67 (0.49–0.90)	1.18 (0.98–1.42)
Age <75 years	0.63 (0.46–0.86)	0.70 (0.57–0.86)
Dabigatran 110 mg BID		
Age ≥75 years	0.88 (0.66–1.17)	1.01 (0.83–1.23)
Age <75 years	0.93 (0.70–1.23)	0.62 (0.50–0.77)
ROCKET-AF		Major bleeding
Age ≥75 years	0.80 (0.63–1.02)	1.11 (0.92–1.34)
Age <75 years	0.95 (0.76–1.19)	0.96 (0.78–1.19)

Prior CAD		

ARISTOTLE		
Prior CAD	0.95 (0.71–1.27)	0.78 (0.62–0.99)
No prior CAD	0.70 (0.56–0.89)	0.64 (0.53–0.77)
RE-LY∗		Major bleeding
Dabigatran 150 mg BID		
Prior CAD/MI	0.75 (0.51–1.10)	0.95 (0.75–1.20)
No prior CAD/MI	0.57 (0.48–0.76)	0.90 (0.80–1.13)
Dabigatran 110 mg BID		
Prior CAD/MI	0.78 (0.58–1.13)	0.88 (0.75–1.10)
No prior CAD/MI	0.90 (0.75–1.20)	0.76 (0.63–0.90)
ROCKET-AF		
Prior MI	0.92 (0.63–1.34)	1.21 (1.02–1.43)
No prior MI	0.87 (0.73–1.04)	n/a

∗HR were visually estimated from primary publication.
